# Nature’s Methods

**Published:** 1877-12

**Authors:** Titus Merritt


### *NATURE'S METHODS.*

We cheerfully give place to the following communication from Dr. Titus Merritt, who has for many years been favorably known as a successful practitioner in a large and highly important class of diseases, and whose favorite remedial agents are those which nature so abundantly supplies. It is always a gratification to us to give publicity to the convictions and experiences of medical men of broad views and extended observation in any department of medical science. EDITOR HALL'S JOURNAL.

DR. GIBBS.—*Dear Sir:*—I have no doubt that nearly all physicians find a good deal to interest them in the pages of HALL'S JOURNAL OF HEALTH. I am free to say that I never glance through a number of this widely known periodical, without learning something. It appears to keep pace with the scientific developments recorded in the strictly professional periodicals, and to so popularize science as to make it palatable to the masses. I read a good many professional journals, but I conceive that I get more real practical good from the pages of HALL'S JOURNAL than from all the rest.

I am charmed to see that you are not afraid to utter encouraging words to the innovators who dare to say that Nature's methods for the alleviation of many forms of physical misery, are often superior to the less kindly devices of man. In looking over your issues for the past few years, I find interesting and valuable articles on the use of Electricity and Ozone, in various diseased conditions; and as these potent agents have been largely employed by me, it has been a source of genuine gratification to observe that your conclusions as to their value differs in no essential particular from my own.

A few weeks since, in conversation with Mr. Edison, the leading electrician of the world, I asked him if he supposed we had nearly reached the end of invention in the realm of electrical science; to which he replied that electrical science was a hundred-acre field, surrounded by a high fence, through the knotholes of which some slight glimpses were being had of the amazing wealth within. I believe this remark is as true concerning the application of electrical currents for the relief of the physical ills of humanity, as in any other direction; and I shall not be surprised to learn that electricity has proved itself to be, when wisely administered, the best stimulant, the best tonic, the best alterative, the best narcotic, the best alleviator of pain, to be fouud in the entire range of thereputic agents.

One thought has long dwelt in my mind, viz., that provision ought to be made for home treatment of the more common diseases, by some potent yet harmless agencies. I have not greatly doubted that the time would come when electricity would be so easily understood and so entirely managable, as to be within the reach of every family. That happy day has not yet dawned, however. This powerful agent is greatly misunderstood and constantly misapplied. Marvellously valuable when wisely used, it becomes a dangerous weapon in the hands of the unskilled.

One great remedial agent I have succeeded in popularizing, and in adapting to home use. This agent is Ozone. In an editorial in the JOURNAL OF HEALTH, you aptly characterize this agent as "active oxygen." It *is* active oxygen; oxygen in its most active state. It is a deadly enemy to all fungoid growths, to all fermentive action, to all septic conditions. Brought in contact with bacteria, they instantly perish. It neutralizes the poison of malaria, and purifies alike contaminated air, and the blood which has been poisoned by its inhalation. This being the case, it is easy to see that those diseases of the throat and lungs which are superinduced by bad air should be relieved by this agent. To adapt it to home use by a simple and cheap appliance, has been my aim, and in this I have succeeded to my entire satisfaction. My inhalers are now largely used, not simply by physicians, but in families; and those who have them at hand, ready for use at a moment's notice, do not suffer from diphtheria, catarrh, bronchitis, hoarseness, or other affections of the air-passages.

I may not trespass upon your time to allude to the many forms of disease which are susceptible of permanent relief by the use of ozone, aided by electricity. I shall be pleased to meet all who would learn more concerning these subjects, at my office, 152 East Fifty-second Street, New York, or to respond without charge to all who may make inquiries by mail. I will even go further than this. I will fully explain my system to all who may call upon me from you, to all readers of the JOURNAL, in fact, and will also give to all such who desire it, one treatment or application free of all charge. Respectfully yours,

TITUS MERRITT.

---

### *GOOD READING.*

Mr. R. W. Rand, of Lyons, Iowa, asks us "What publications do you consider essential in a family where there are children of various ages?" We reply: By all means, as foundation stones, Webster's Unabridged Dictionary and Zell's Encyclopedia. Then should come Appleton's Popular Science Monthly, and the supplement thereto; Harper's Magazine, Weekly and Bazar; Scribner's Magazine and St. Nicholas. We do not suggest HALL'S JOURNAL OF HEALTH because we know Mr. Rand to be a most sensible gentleman, who values the health of the family above all things, and accordingly fully appreciates the value of the JOURNAL, which teaches mankind how to preserve it. There are many other publications that it is desirable to have, but the above would certainly be our first choice.

